# Supplementary material for: Information based explanation methods for deep learning agents—with applications on large open-source chess models
Source: Sci Rep. 2024 Aug 30;14:20174. doi: 10.1038/s41598-024-70701-2 (PMC11364554; doi:10.1038/s41598-024-70701-2)
Supplement: Supplementary file 1 — Supplementary Information. [file 41598_2024_70701_MOESM1_ESM.pdf]

Supplementary Table S1. Input description of each input plane used for training our custom model with appended II-map module. Adapted for use from Hammersborg [5].

Plane number	Description
**0 – 5**	One plane for each piece-type for the player to move.<br>(in the order of pawn, knight, bishop, rook, queen, king)
**6 – 11**	One plane for each piece-type for the opposing player.<br>(in the order of pawn, knight, bishop, rook, queen, king)
**12 – 15**	Kingside, queenside castling rights for both players<br>(not used in the presented variants)
**16**	If Black is the player to move.
**17**	Counter of the amount of moves since the last of<br>capturing- or pawn-move. Used for the 50 move rule.<br>(When no capturing- or pawn-moves have been<br>made during the last 50 moves, any player can claim a draw.)
**18**	All zeros.
**19**	All ones.

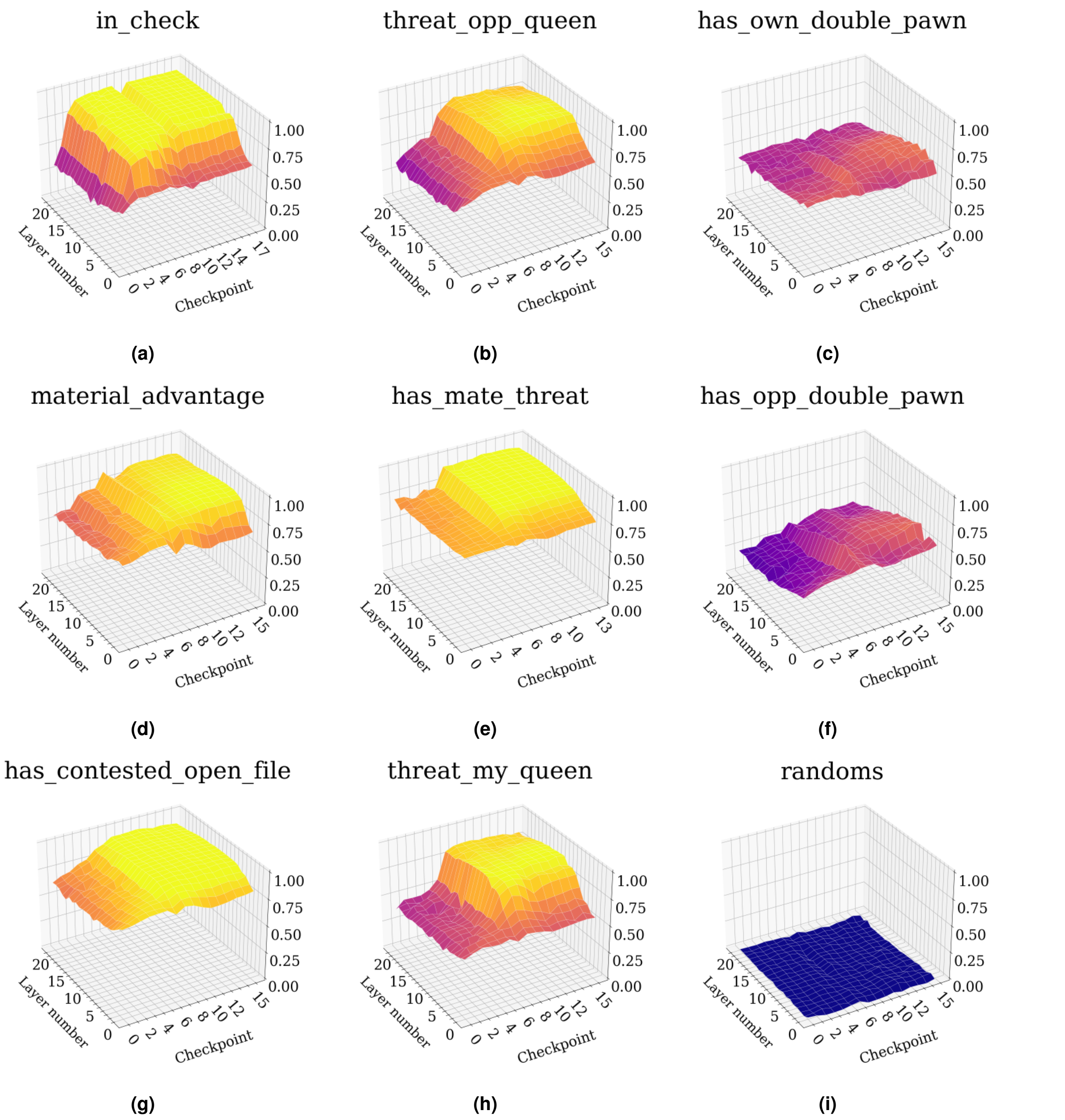

Supplementary Figure S1. Concepts modelled by the 8x8 chess agent, showing the model's ability to detect (a) whether the player to move is in check, (b) whether the opponent's queen is under threat, (c) whether it has a double-pawn, (d) whether the player to move has a material advantage, (e) whether the opponent is currently presenting a mate-threat, (f) whether the opponent has a double-pawn, (g) whether both players contest an open file on the board, and (h) whether the player to move's queen is threatened, and (i) being a sanity check performed on a data set of random labels.

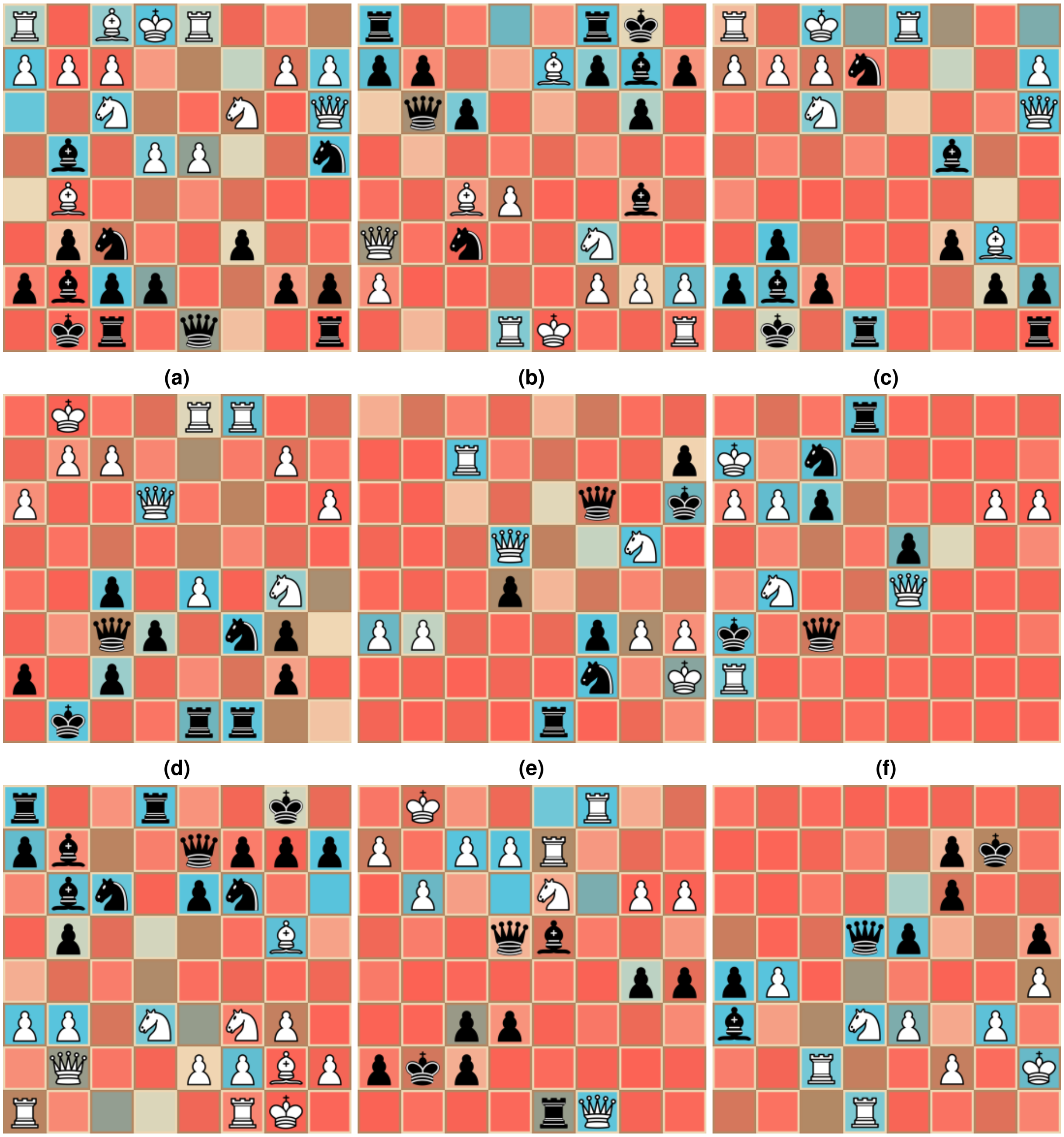

(a) (b) (c) (d) (e) (f) (g) (h) (i)

Supplementary Figure S2. Masks generated for a set of positions from well-known chess games for the models described in Sec. 3.2.3. The positions in Figs. (a) to (c) show positions from "The Game of the Century", Figs. (d) to (f) show positions from the first game between Garry Kasparov and IBM's DeepBlue, and Figs. (g) to (i) show positions from the sixth game from the 2021 World Championships.
